# Study on the Structure-Property Dependences of Rigid PUR-PIR Foams Obtained from Marine Biomass-Based Biopolyol

**DOI:** 10.3390/ma13051257

**Published:** 2020-03-10

**Authors:** Paulina Kosmela, Aleksander Hejna, Jan Suchorzewski, Łukasz Piszczyk, Józef Tadeusz Haponiuk

**Affiliations:** 1 Department of Polymer Technology, Chemical Faculty, Gdansk University of Technology, Narutowicza Str. 11/12, 80-233 Gdansk, Poland; aleksander.hejna@pg.edu.pl (A.H.); lukpiszc@pg.gda.pl (Ł.P.); jozhapon@pg.gda.pl (J.T.H.); 2 RISE Research Institutes of Sweden, Infrastructure and Concrete Technology, Material Design, 501-15 Borås, Sweden; jan.suchorzewski@ri.se; 3 Department of Civil and Material Engineering, Faculty of Civil and Environmental Engineering, Gdansk University of Technology, Narutowicza Str.11/12, 80-233 Gdansk, Poland

**Keywords:** bio-based polyol, biomass liquefaction, rigid polyurethane-polyisocyanurate foams, structure-property relationship, isocyanate index

## Abstract

The paper describes the preparation and characterization of rigid polyurethane-polyisocyanurate (PUR-PIR) foams obtained with biopolyol synthesized in the process of liquefaction of biomass from the Baltic Sea. The obtained foams differed in the content of biopolyol in polyol mixture (0–30 wt%) and the isocyanate index (I_ISO_ = 200, 250, and 300). The prepared foams were characterized in terms of processing parameters (processing times, synthesis temperature), physical (sol fraction content, apparent density) and chemical structure (Fourier transform infrared spectroscopy), microstructure (computer microtomography), as well as mechanical (compressive strength, dynamic mechanical analysis), and thermal properties (thermogravimetric analysis, thermal conductivity coefficient). The influence of biopolyol and I_ISO_ content on the above properties was determined. The addition of up to 30 wt% of biopolyol increased the reactivity of the polyol mixture, and the obtained foams showed enhanced mechanical, thermal, and insulating properties compared to foams prepared solely with petrochemical polyol. The addition of up to 30 wt% of biopolyol did not significantly affect the chemical structure and average cell size. With the increase in I_ISO_, a slight decrease in processing times and mechanical properties was observed. As expected, foams with higher I_ISO_ exhibited a higher relative concentration of polyisocyanurate groups in their chemical structure, which was confirmed using principal component analysis (PCA).

## 1. Introduction

Algae biomass is commonly used around the world on an industrial scale, mainly for consumer, industrial, pharmaceutical, and cosmetology purposes. Algae have been used in the food industry for many years as a rich source of low-calorie nutrients [1,2,3]. In the cosmetics industry, mainly dried, freeze-dried algae, and algae extracts are used [4]. Algae biomass is characterized by a high content of various biologically active compounds, among others with antibacterial, antiviral, antifungal, and anti-inflammatory effects [5,6,7,8]. Due to its renewable nature, interest in using algae has increased in recent years, including in the energy sector [9] and the plastics industry [10].

Plastics with algae biomass can be obtained in many ways. The main methods of preparation include direct and indirect methods. Direct methods rely on mixing algae biomass with a polymer matrix to obtain biocomposites. Much research has been done on the use of algae biomass as a reinforcement in composites [11,12,13]. Jang et al. [14] received biocomposites using polypropylene (PP) and pre-processed algae Laminaria japonica (BA) and Enteromorpha (GA). Algae pre-treatment included treatment with ethanol at 78 °C for 3 h, followed by washing with ethanol or acetone. Other samples were treated with 3 wt% of sulfuric acid at 121 °C for 2 h. In the last stage of preparation, BA or GA was washed with distilled water, dried at 100 °C for 24 h, and milled. Biocomposites were obtained by the compression molding method. PP was evenly mixed with BA or GA and dried in a convection oven at 100 °C for 24 h. PP/GA biocomposites showed higher thermal stability and impact resistance than PP/BA ones. It was also found that sulfuric acid-treated GA biocomponents showed the best thermal and mechanical properties.

Indirect methods consist of obtaining plastics from the algae biomass used as an intermediate in different processes. An example of the indirect use of algae is the extraction of alginate, well described in the literature, and its use in the synthesis of polyurethane [15,16,17,18]. Another indirect method is the use of algae oil in the synthesis of polyurethane foams [19,20,21]. Pawar et al. [22] modified oil from chlorella microalgae and used it to obtain rigid polyurethane foams. The modification consisted of oil epoxidation and opening of the oxirane ring using lactic acid and ethylene glycol. Oil epoxidation was carried out in solution using peracetic acid and hydrogen peroxide for 6–8 h with a yield of 90–94%. In the next stage, the epoxidized oil was mixed with lactic acid (1:6 molar ratio) or ethylene glycol (1:10 molar ratio) to obtain biocomponents with active hydroxyl groups. Rigid polyurethane foams were obtained using polyol solely from algae oil and compared with their petroleum-based analogs. Foams obtained with the use of algae oil are characterized by a higher content of closed cells and similar thermal stability compared to petrochemical foam. Although the presented procedure resulted in foams showing satisfactory properties, the synthesis of applied biopolyol consisted of several steps including extraction of oils from microalgae, its epoxidation and reaction with lactic acid, as well as purification steps. Furthermore, it required the use of significant amounts of solvents, which cannot be considered environmentally friendly. 

In our previous work [23], we proposed a method of direct liquefaction of marine biomass from the Baltic Sea, which was only dried before processing. Moreover, to enhance the eco-friendly character of the process, we applied waste material (crude glycerol) as one of the solvents in the process. The developed procedure provided a method of utilization of marine biomass from the Baltic Sea, which especially during the summer is a troublesome waste. This paper presents the method of preparation and the characteristics of rigid polyurethane-polyisocyanurate foams obtained using biopolyol resulting from the abovementioned liquefaction process. Rigid polyurethane-polyisocyanurate (PUR-PIR) foams were obtained by a one-step method, by replacing up to 30 wt% petrochemical polyol with biopolyol. Processing, chemical structure, and morphology, as well as mechanical and thermal properties were characterized.

## 2. Materials and Methods 

### 2.1. Materials

**Figure 1:**
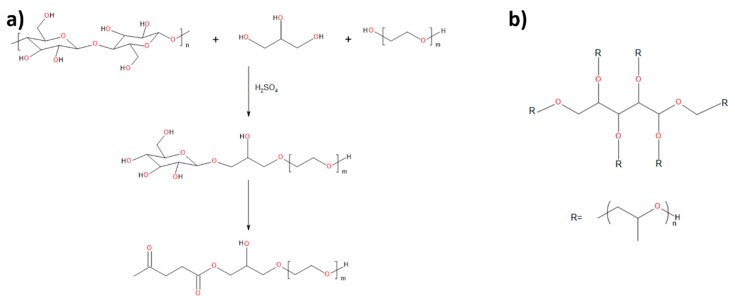
(**a**) proposed course of the main reaction occurring as a result of liquefaction of biomass; (**b**) chemical structure of Rokopol®RF551.

For the preparation of rigid PUR-PIR foams, LB biopolyol obtained via marine biomass (10 wt%) liquefaction with a mixture of crude glycerol and poly(ethylene glycol) (90 wt%) was applied. The applied biomass consisted of Enteromorpha macroalgae and Zostera marina seagrass in the ratio of 1 to 9. The proposed structure of biopolyol is presented in Figure 1, while its synthesis and properties were described in another work [23]. Rokopol®RF551 (polyoxyalkylene polyhydric alcohol), obtained from the PCC Group (, Brzeg Dolny, Poland), was used as the petrochemical polyol. The selected properties of both polyols are shown in Table 1. The isocyanate component was polymeric methylene diphenyl-4,4’-diisocyanate (pMDI) with a free isocyanate groups (NCO) content of 31.5% from BASF (Ludwigshafen, Germany). The solution of potassium acetate in ethylene glycol (AC) (PC CAT® TKA30 from Performance Chemicals (Buchholz in der Nordheide, Germany)), 75 wt% solution of potassium octoate in diethylene glycol (Dabco K15), 33 wt% solution of triethyl diamine in dipropylene glycol (Dabco33LV from Air Products (Allentown, PA, USA)), and dibutyltin dilaurate (DBTDL) from Sigma Aldrich (Saint Louis, MO, USA) were applied as catalysts. Tegostab B8465 from Evonik Industries AG (Essen, Germany) was applied as a surfactant (SPC), and n-pentane from Lachner was used as a blowing agent. Trichloropropyl phosphate (TCPP) was also added as a flame retardant, which also reduced the viscosity of the polyol mixture (TCPP was characterized with a viscosity of 61–89 mPa·s) from LANXESS Deutschland GmbH (Koln, Germany).

### 2.2. Preparation of Rigid PUR-PIR Foams

Rigid PUR-PIR foams were obtained on a laboratory scale using the single-step method from the two-component system. The impact of the isocyanate index (I_ISO_) on the processing of polyurethane systems and the performance of the resulting PUR-PIR foams was investigated. Three values of I_ISO_ were applied, basing on literature reports and our previous works: 200, 250, and 300 [24,25]. Component A consisted of the proper amounts of Rokopol®RF551 and biopolyol LB, catalysts, surfactant, flame retardant, and blowing agent. Component B was isocyanate. Both components were mixed in a polypropylene cup with a mechanical stirrer at 2000 rpm and poured into the open mold. Foams were cured for 24 h at 60 °C and then conditioned at room temperature for 24 h. Table 2 contains the details of foam formulations. 

The performance of rigid PUR-PIR foams, as well as all other porous materials, is closely related to their apparent density. Therefore, it is essential to analyze the materials characterized by a similar level of this parameter. In Table 2, there are also presented values of apparent density, which for all samples was in the range of 49.2–53.2 kg/m^3^. It was possible by the adjustment of foams’ formulations and varying content of potassium catalyst and foaming agent. The rise of the isocyanate index increased the amount of these components required for providing the desired level of apparent density, which was also observed in other works [26].

### 2.3. Characterization of Polyurethanes

#### 2.3.1. Evaluation of the Foaming Process

For all the samples, the following processing times were determined: start time (from the beginning of mixing to the start of volumetric expansion), rise time (time of volumetric expansion), and the tack-free time (from the end of volumetric expansion to the point when the surface stopped being tacky to the touch). After pouring the reaction mixture into the mold, the temperature inside the foam was measured by a thermocouple.

#### 2.3.2. Fourier-Transform Infrared Spectroscopy 

FT-IR spectrophotometric analysis was performed to determine the structure of the bio-based polyol and rigid PU-PIR foams. The analysis was performed at a resolution of 4 cm^−1^ using a Nicolet 8700 apparatus (Thermo Electron Corporation, Waltham, MA, USA) equipped with a snap-Gold State II, which allowed for making measurements in the reflection configuration mode. Principal component analysis (PCA) was applied to analyze the results with MATLAB software (MathWorks, Natic, MA, USA).

#### 2.3.3. Apparent Density

The apparent density of samples was calculated in accordance with EN ISO 845:2000 [27], as a ratio of the sample weight to the sample volume (g/cm^3^). The cylindrical samples were measured with a slide caliper with an accuracy of 0.1 mm and weighed using an electronic analytical balance with an accuracy of 0.0001 g.

#### 2.3.4. Sol Fraction Content

To determine the sol fraction content in prepared foams, 0.2 g samples were placed in xylene for 72 h at room temperature. Then, samples were taken out and dried until constant weight. Sol fraction content was calculated according to the following Formula (1):

(1) $$X=\frac{m_{1}-m_{2}}{m_{1}}\;\cdot 100\%$$

where: *m*_1_ is the initial mass of the sample and *m*_2_ is the mass after extraction and drying. 

#### 2.3.5. Compressive Strength

The compressive strength of rigid foams was estimated in accordance with EN ISO 844:2007 [28]. The cylindrical samples with dimensions of 20 mm × 20 mm (height and diameter) were measured with a slide caliper with an accuracy of 0.1 mm. The compression test was performed on a Zwick/Roell 1000 N testing machine (ZwickRoell GmbH & Co. KG., Ulm, Germany) at a constant speed of 10%/min until reaching 15% deformation.

#### 2.3.6. Thermal Conductivity Coefficient

The thermal conductivity of foams was tested with Lambda 2300 heat flow meter (Holometrix, Bedford, MA, USA) according to the ASTM C518 standard [29]. The average temperature of analysis was 10 °C and the temperatures of the lower and upper plates 0 and 20 °C, respectively. Three samples with a size of 300 mm × 300 mm × 50 mm were tested for each composition. 

#### 2.3.7. Thermogravimetric Analysis

To evaluate the thermal stability of materials, thermogravimetric analysis (TGA) was performed on 5 mg samples using a NETZSCH TG 209 F3 apparatus (NETZSCH-Gruppe, Selb, Germany) under a nitrogen atmosphere in the temperature range from 40 to 800 °C and at a heating rate of 20 °C/min.

#### 2.3.8. Dynamic Mechanical Analysis 

The dynamic mechanical analysis (DMA) was performed using the DMA Q800 TA Instruments apparatus (TA Instruments Inc., New Castle, DE, USA). Samples were analyzed in compression mode with a frequency of 1 Hz and an amplitude of 20 μm. Measurements were performed for the temperature range from 35 to 270 °C with a heating rate of 4 °C/min. Samples were cylindrically shaped with dimensions of 6 mm × 18 mm.

#### 2.3.9. Micro-Computed Tomography

Foams were analyzed with a 3D Skyscan 1173 X-ray microscope (Bruker, Kontich, Belgium) with a resolution of 9 μm and a rotation of 180°. The energy of X-ray radiation was 35 kV, a current of 175 μA, and the sample exposition time was 500 ms. A rotation step of 0.2° was applied for high precision. Skyscan Nrecon software (6.0 Skyscan, Kontich, Belgium) was used to obtain 3D images, which were then analyzed with CTAn software. The X-ray microscope used in this study represented the new generation of apparatus with high resolution [30,31].

## 3. Results and Discussion

### 3.1. Evaluation of the Foaming Process 

The values of the start time and tack-free time for all samples equaled 10 and 0 s, respectively. this means that when foams reached their maximum volume, their surface was already not sticky. Therefore, in Table 3 are presented only the values of the rise time and maximum temperatures reached by the foams during the reaction. It can be seen that the rise time was slightly reduced by the increase of biopolyol content in foams’ formulations, which could be related to the lower viscosity of biopolyol LB compared to Rokopol®RF551. Furthermore, a significant increase of the maximum temperature reached during foaming was noted, which could be associated with the enhanced reactivity of the systems with a higher content of biopolyol. The increased reactivity of biopolyol was associated with its chemical structure (higher hydroxyl number L_OH_ = 650 mg KOH/g) and higher viscosity polyols (η = 2236 mPa·s) compared to petrochemical polyol. Lower viscosity (greater mobility of chains, functional groups) and the higher concentration of reactive groups caused acceleration of the reaction of allophanate crosslinks, urethane, and urea linkages. A similar effect was noted in other works [32].

The values of rise time and T_MAX_ were also affected by the isocyanate index. It could be seen that shortening of the rise time was noted, but the maximum temperature was reduced, when I_ISO_ was increased. Such an effect was probably associated with the increased loading of the blowing agent for higher isocyanate content, which was necessary to provide a similar level of the foams’ apparent density. The applied *n*-pentane was the physical blowing agent, so it required heat to evaporate and generate a porous structure of PUR-PIR foam. Moreover, the reduction of T_MAX_ could be related to the lower enthalpy for the isocyanate trimerization reaction (ΔH = 80 kJ/kmol), compared to the generation of urethane bonds (ΔH = 105 kJ/kmol) [33]. 

### 3.2. Microstructure of Rigid PUR-PIR Foams

**Figure 2:**
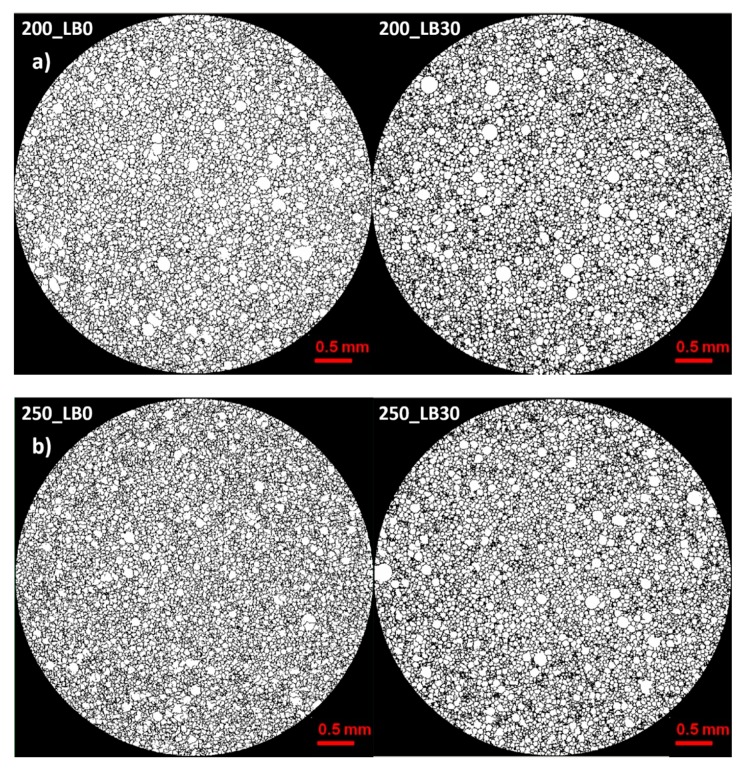

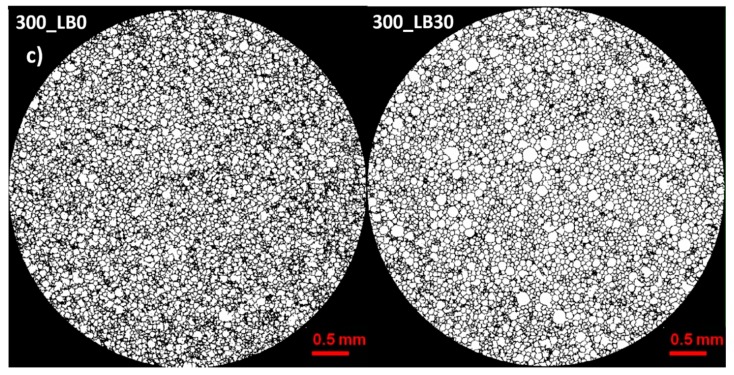
2D images of PUR-PIR foams: (**a**) I_ISO_ = 200, (**b**) I_ISO_ = 250, (**c**) I_ISO_ = 300.

Micro-computed tomography (micro-CT or μCT) is a technique that enables three-dimensional imaging of a sample and the generation of virtual 3D models with the use of X-ray radiation. In Figure 2 are presented two-dimensional cross-sections of PUR-PIR foams prepared solely from petrochemical polyol and with a 30 wt% share of biopolyol. A slight decrease of the average cell size was noted, which could be associated with the presence of solid particles in the applied biopolyol. The applied biopolyol was obtained with 78% biomass conversion, as mentioned in our previous work [23]. Therefore, considering the applied formulations, foams with an isocyanate index of 200, 250, and 300 contained 1.8, 1.5, and 1.4 wt% of solid particles, which might act as nucleating agents, simultaneously reducing the average cell diameter. A similar effect was noted by Zhang et al. [34]. Moreover, the values of porosity are presented, which were calculated as the ratio of the total cell volume and the volume of the measured sample. It could be seen that the porosity was slightly decreasing with the addition of biopolyol, which was probably associated with the lower viscosity of biopolyol LB compared to commercial Rokopol®RF551 (see Table 1). Such an effect was related to the lower resistance of the foaming mixture to the cells’ coalescence. The more viscous polyol mixture showed a higher ability to trap gas inside [35]. The effect was very significant in the case of the work presented by Fan et al. [36]; however, they substituted conventional polyol with a viscosity of 9000 cP with a soy-based one, which showed a value of 31,351 cP. In the case of presented work, differences in the polyols’ viscosity were not so significant. A similar effect was observed in the work of Ciecierska et al. [37], who analyzed the impact of the viscosity of a polyol mixture on the cellular structure of rigid PUR foams. Their results showed that the increase of the viscosity caused an increase in the porosity, without significant changes in cell diameters. Nevertheless, such changes in morphology caused the deterioration of the mechanical performance. 

**Figure 3:**
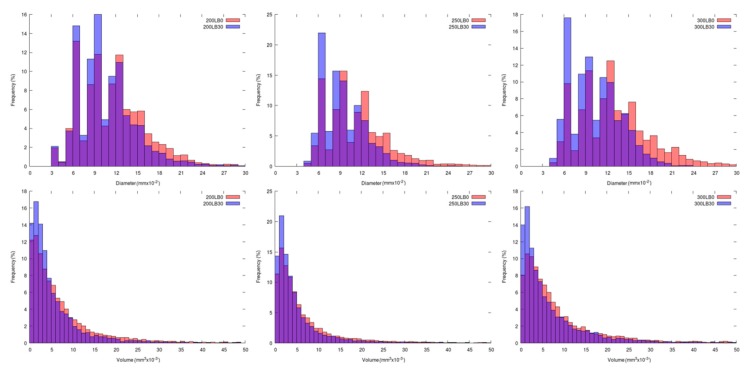
Histograms showing the distribution of the pore size (upper line) and pore volume (lower line) of PUR-PIR foams for different isocyanate indexes.

In Table 4 are presented the parameters of the foams’ cellular structure. It could be seen that the incorporation of biopolyol resulted in the reduction of the average cell diameter and volume. Moreover, the increase of I_ISO_ resulted in the enhancement of the microstructure homogeneity, which was confirmed by the decrease of the standard deviation and the sharper shape of the histograms presented in Figure 3. For all samples containing 30 wt% of biopolyol, a significant increase in the content of cells with a diameter of ~60 μm was observed. Regarding the influence of I_ISO_, the highest homogeneity of structure and the lowest values of cell size and volume were noted for the ratio of isocyanate to hydroxyl groups equal to 250. Moreover, in Table 5 are presented 3D images showing the distribution of cells with a particular volume inside foams.

In Table 6 are presented the thermal insulation properties of prepared foams. The thermal conductivity coefficient (λ) and thermal resistance (R) are crucial for determining the possibility of the application of PUR-PIR foams as insulation materials. For these materials, the λ coefficient was composed of four components λ_gas_, λ_PUR-PIR_, λ_radiation_, and λ_convection_ [38]. The share of these components was strictly correlated with the cellular structure of the foams. With the increase of apparent density, the thermal conductivity of a solid part of the foam would have a more significant influence on the total λ value. Therefore, a low density was very beneficial for the performance of thermal insulation materials since the λ values for solid PUR-PIR, CO_2_, and air were 220.0, 15.3, and 24.9, respectively [25]. Other structural parameters, such as the cell size, also showed a significant impact on the thermal conductivity of foamed materials. According to Glicksman [39], λ_radiation_ was proportional to cell diameter and inversely proportional to the density of the foam and solid polymer. Randall and Lee [40] proved that the decrease in average cell diameter from 600 to 250 μm resulted in a drop in the thermal conductivity coefficient by almost 50%. Moreover, to show the lowest possible value of the λ parameter, foams should be characterized by a high content of closed cells, which reduced λ_convection_ by trapping gas inside the foam. Volatile hydrocarbons, commonly applied as blowing agents for PUR-PIR foams, showed thermal conductivity coefficient ~1.5 and ~2.5 lower than CO_2_ and air.

It could be seen that the incorporation of biopolyol into the foams’ formulation resulted in the reduction of the average cell size, which was followed by a drop of the thermal conductivity coefficient. A similar effect was observed in our other works [24,25]. Changes of the isocyanate index did not result in significant differences in the thermal conductivity of prepared foams.

### 3.3. Physico-Mechanical and Thermal Properties Analysis

In Table 7 are presented the values of the sol fraction, compressive strength, and glass transition temperature (T_g_) of foams determined from the temperature dependence of the loss tangent. The addition of biopolyol into the formulations of PUR-PIR foams reduced the content of the sol fraction. This was associated with a decrease of the non-crosslinked fraction content, which suggested a higher crosslink density of prepared foams. The decrease of the sol fraction content was also affected by the increase of I_ISO_, which confirmed other research works [41]. Except for the primary condensation reaction, which led to the generation of urethane groups, at higher values of I_ISO_, additional reactions may take place. They included the generation of allophanate and biuret groups or trimerization of isocyanates leading to the generation of polyisocyanurates. All of these groups could noticeably affect the level of crosslink density of PUR-PIR foams [42]. 

In Table 7 are also presented the values of the compressive strength of rigid PUR-PIR foams, measured in two directions, parallel and perpendicular to the rise direction. It was noted that the mechanical performance of the foams was significantly affected by their cellular structure, which was also proven in other works related to this type of material [43,44]. For the analyzed materials, the anisotropy of the mechanical properties was associated with the elongation of cells in the direction of the foam rise (see the 3D images in Table 5). More significant differences between compressive strength parallel and perpendicular to the rise direction were noted for lower contents of biopolyol. This effect was not observed for the highest value of I_ISO_, which could be related to the increased stiffness of the structure. Moreover, the increase of the isocyanate index caused a slight reduction of compressive strength, which was also observed by other researchers [45]. Such an effect was associated with the higher friability of the foams’ structure, which was already noted by Modesti and Lorenzetti [42].

Except for the decrease of anisotropy in the mechanical properties, a general enhancement of compressive strength was observed for the increase of biopolyol content. Such an effect was related to the reduced values of the sol fraction, suggesting the enhanced crosslink density of modified foams, as well as changes in the cellular structure (a decrease of the cell size and porosity). Generally, a decrease of the cell size with a similar level of the materials’ porosity resulted in the enhancement of the mechanical performance [46]. 

The glass transition temperature of the foams was significantly affected by the changes in the foams’ formulations. The increase of the biopolyol content and isocyanate index led to the rise of T_g_, which indicated the stiffening of the material, and together with a drop of the sol fraction content, indicated an increase of crosslink density. Ivani et al. [47] stated that for rigid PUR-PIR foams, an increase of T_g_ with the addition of biopolyol was associated with the lower flexibility of macromolecular chains compared to the conventionally applied petrochemical polyols. 

### 3.4. Thermogravimetric Analysis

**Figure 4:**
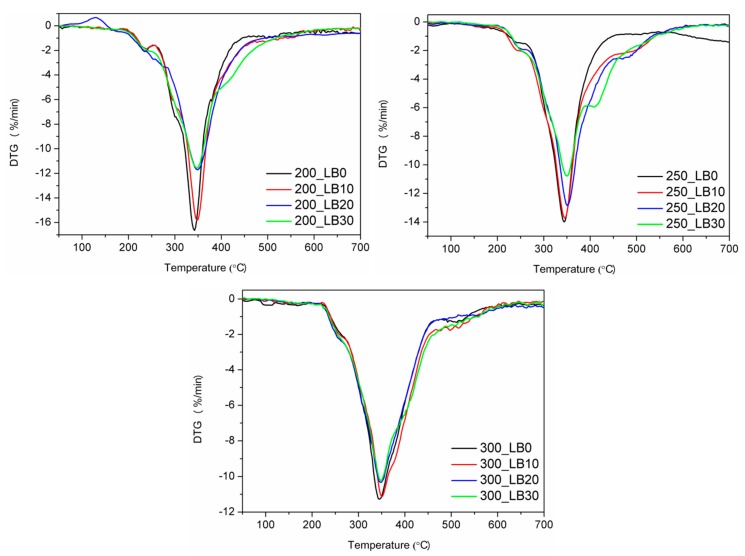
DTG curves of rigid PUR-PIR foams.

In Figure 4 and Table 8 are presented the results of the thermogravimetric analysis. The thermal decomposition of polyurethanes is a very complicated process, due to its complex structure, especially when also polyisocyanurate groups are present. Generally, it could be seen that the increase of biopolyol content shifted the onset of thermal degradation, measured by the temperature of 2 wt% mass loss, towards higher temperatures. The only exception was observed for I_ISO_ of 200 and the highest content of biopolyol. It could be considered as very beneficial because numerous literature reports pointed to the deterioration of thermal stability caused by the application of biopolyols [48].

Differential thermogravimetric curves indicated the three-step decomposition process for all samples. The first step, observed at 220–280 °C, was associated with the decomposition of TCPP (degradation temperature of 224 °C), as well as the decomposition of soft segments. During the second step, with the temperature of the maximum degradation rate of 350 °C, hard segments of polyurethane were decomposed, and amines and carbon dioxide were formed [49]. For sample 250_LB30, an additional peak around 410 °C was observed, which was associated with biopolyol decomposition [32]. The third step of degradation, in the range of 430–570 °C, was related to the thermolysis of organic residues from previous steps [50]. The incorporation of synthesized biopolyol into foams’ formulations caused a shift of peaks on DTG curves towards higher temperatures, which confirmed the enhancement of thermal stability.

### 3.5. Infrared Spectroscopy and Principal Component Analysis 

#### 3.5.1. FTIR Spectra Analysis

**Figure 5:**
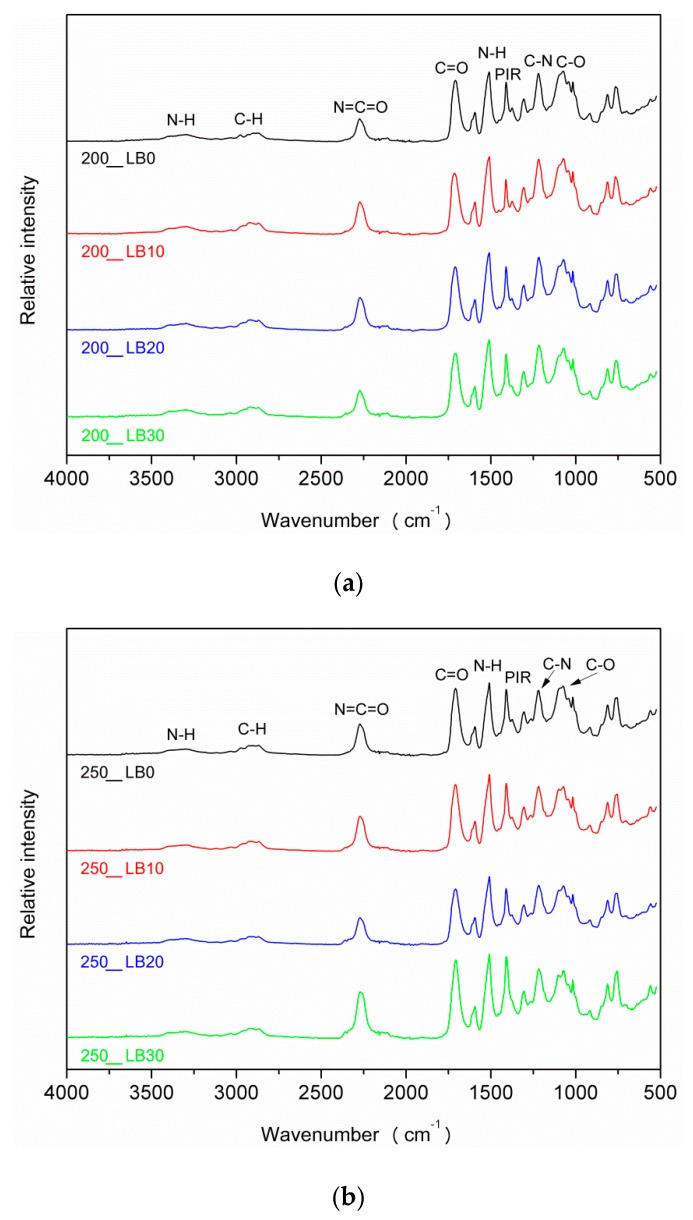

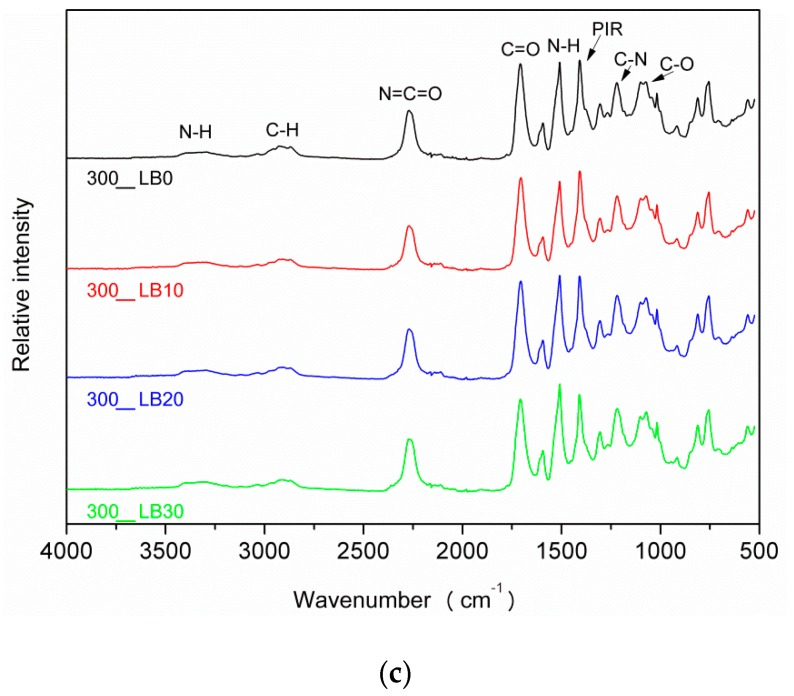
FTIR spectra of PUR-PIR foams: (**a**) I_ISO_ = 200, (**b**) I_ISO_ = 250, (**c**) I_ISO_ = 300.

The analysis of FTIR spectra presented in Figure 5 did not indicate qualitative changes in the chemical structure of prepared PUR-PIR foams after the addition of biopolyol. Absorption bands observed in the range of 3290–3320 cm^−1^ were attributed to the stretching vibrations of N–H bonds in the urethane groups. Signals for bending vibrations of these bonds were noted at 1510–1520 cm^−1^ [51]. Bands at 1200–1215 and 1705–1715 cm^−1^ related to stretching vibrations of C–N and C=O bonds, respectively, confirmed the presence of urethane groups [52]. Signals present in the range of 1410–1415 cm^−1^ were attributed to the presence of isocyanurate rings generated during the trimerization of isocyanate groups [53]. Around 2260–2280 cm^−1^, there were noted peaks, which indicated the presence of free isocyanate groups, also associated with relatively high values of applied I_ISO_ [54].

Moreover, around 2860–2870 and 2960–2975 cm^−1^, there were observed signals related to the symmetric and asymmetric stretching vibrations of C–H bonds in macromolecular chains of applied polyols. Multiplet bands in the range of 1000–1090 cm^−1^ were attributed to the vibrations of C–O bonds in the ester and ether groups present in the structure of polyols [55].

**Figure 6:**
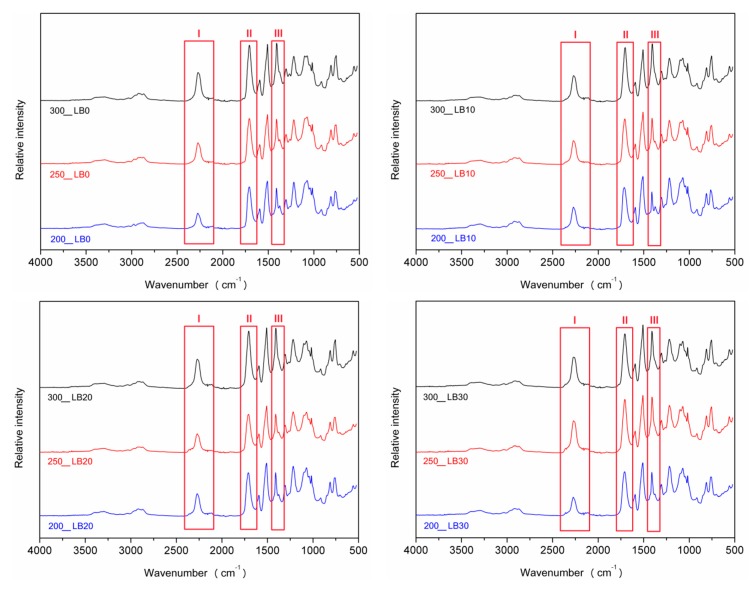
FTIR spectra of foams depending on I_ISO._

In Figure 6 is presented the impact of I_ISO_ on the FTIR spectra of prepared PUR-PIR foams. The increase of the peaks’ magnitude for vibrations of isocyanate groups, carbonyl bonds, and polyisocyanurate rings, marked as I, II, and III, respectively, was noted. As mentioned above, their intensity was noticeably increasing with the rise of I_ISO_ [56].

#### 3.5.2. Principal Component Analysis 

**Figure 7:**
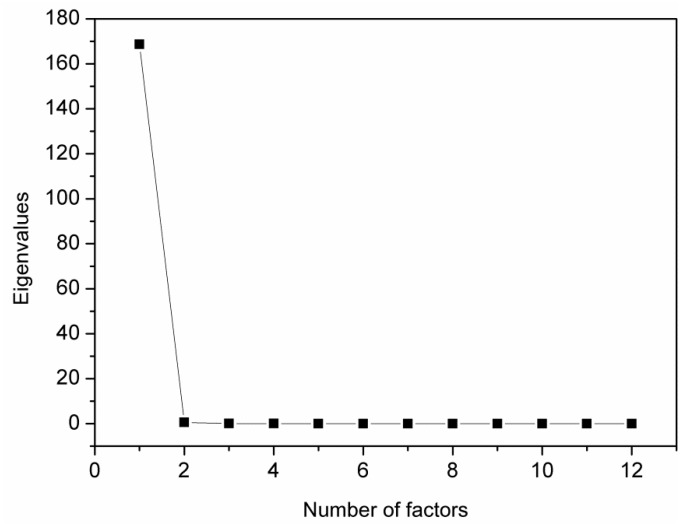
Eigenvalues for 12 factors (12 spectra of PUR-PIR foams).

To determine the content of polyisocyanurate and polyurethane components in the foams’ structure, an analysis of the main factors (PCA) was performed using the Malinowski spectral isolation algorithm. The use of PCA allowed reducing the number of variables, the interpretation of the relationships between components, and the graphical presentation of the configuration of compared variables. The analysis of the main factors for the FTIR spectra of PUR-PIR foams indicated two main factors affecting them, which is shown in Figure 7.

**Figure 8:**
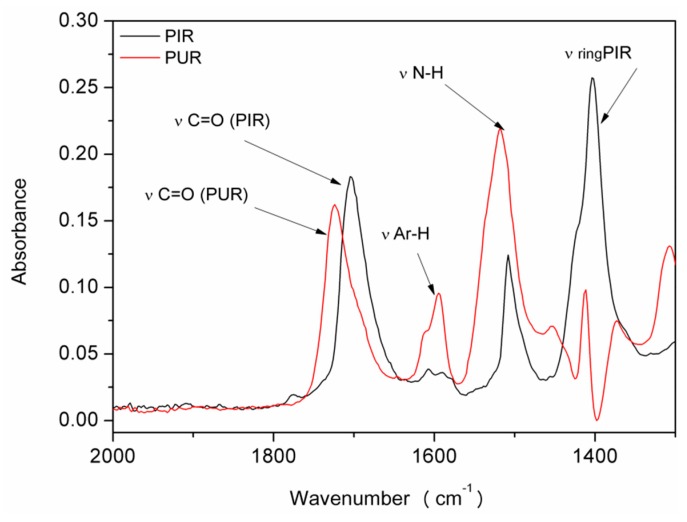
Spectrum of the main factors: PUR and PIR.

After determining the number of principal components, the spectra of pure components were isolated using the Malinowski spectra algorithm. It was found that the two components were: the spectrum derived from PUR and from PIR, which are shown in Figure 8.

In the presented range of wavenumbers of 1300–2000 cm^−1^, one could notice the separation of the absorbance band for the C=O bond, for individual components. Other researchers also observed the shift of the band associated with PIR towards lower wavenumbers. Xu et al. [57] found out that, in the PIR foams, the carbonyl group bonded to the isocyanurate ring showed a coupling effect, which resulted in a shift in the absorbance peak towards the lower values. The other bands presented in Figure 8 are characteristic of individual components. Absorbance bands in the range of 1595 cm^−1^ in polyurethane confirmed the presence of aromatic rings derived from isocyanate [58]. The lack of this absorbance band in the case of PIR foam with the simultaneous appearance of the band at 1403cm^−1^ confirmed the formation of isocyanate group trimerization products [57].

**Figure 9:**
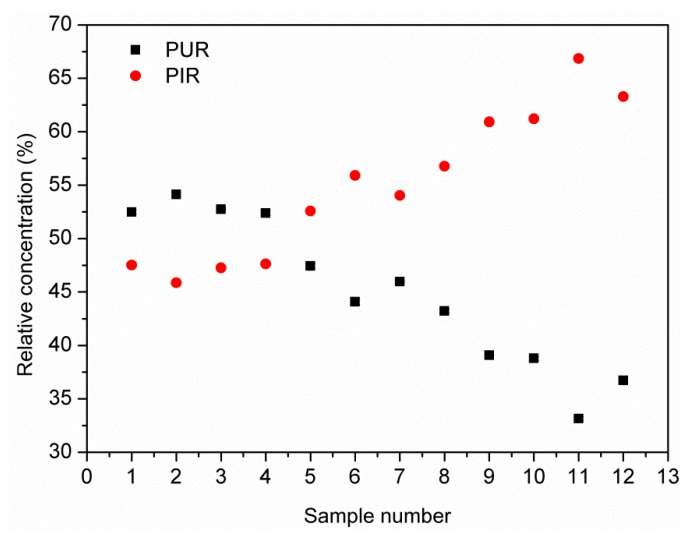
Relative concentrations of major factors.

To determine the percentage content of particular components in PUR-PIR foams, relative concentrations were calculated according to the following formula (2):

C_w,a_ = C_a_ / (C_a_ + C_b_) (2)

where: C_w,a_ is the relative concentration of Component a in the mixture; C_a_ is the concentration of Component a; and C_b_ is the concentration of Component b. In Figure 9 are presented the relative concentrations of PUR and PIR for the analyzed foams. Samples 1–4 were foams obtained with I_ISO_ equal to 200 with biopolyol content from 0 to 30 wt%, while Samples 5–8 and 9–12 were obtained with I_ISO_ of 250 and 300, respectively.

The data presented in Figure 9 indicated that the increase of the isocyanate index resulted in the increase of relative concentration of polyisocyanurate structures in foams. It could be seen that the concentrations of PUR and PIR parts of foams were strictly related to each other. Their values were strongly dependent on the isocyanate index because only when isocyanates were used in excess, a significant amount of polyisocyanurate rings could be generated. Moreover, the incorporation of biopolyol resulted in an increase of PIR content, which could partially contribute to the decrease of sol fraction content and stiffening of the material, which resulted in the rise of T_g_. Although the presented results were probably different from the real ones, because, in the case of such materials like PUR-PIR foams, FTIR should not be considered as a quantitative technique, they provided exciting insight into the analysis of the obtained properties of the foams. 

## 4. Conclusions

The paper presented the method of the preparation and characterization of rigid PUR-PIR foams produced from biopolyol obtained by liquefaction of marine biomass. The foams were obtained with varying contents of biopolyol (0, 10, 20, and 30 wt%) and the isocyanate index (200, 250, and 300). Both factors affected the foaming process. A decrease in the rise time and an increase of the maximum temperature during synthesis were observed for the increasing content of biopolyol independently of I_ISO_. This was due to the greater reactivity of the systems containing biopolyol. Furthermore, a decrease in the maximum temperature was observed with an increase in I_ISO_, due to the lower thermal effect of isocyanate trimerization compared to the enthalpy of polyurethane formation. All foams were characterized by apparent density in the range of 49–54 kg/m^3^. Computer microtomography was applied to analyze the microstructure of foams containing 0 and 30 wt% of biopolyol. The average values of the cell diameter and volume of all foams were in the range of 95–131 µm and 5.4–11.0 mm^3^·10^−3^, respectively. Based on the obtained histograms, it was found that the foams containing biopolyol were characterized by a higher content of cells smaller than 60 µm. All foams showed relatively low thermal conductivity λ in the range 24.69–26.98 mW/m·K. Along with the increase in biopolyol content, a decrease in λ by 2.29, 1.24, and 1.26 mW/m·K was observed for I_ISO_ = 200, 250, and 300, respectively. The addition of biopolyol caused enhanced cross-linking of PUR-PIR foams, which was evidenced by a decrease in the amount of sol fraction, an increase in the glass transition temperature, and an increase in compressive strength. To determine the effect of cellular structure on mechanical strength, rigid foams were compressed in two directions, perpendicular and parallel to the rise of foam. Based on the thermogravimetric analysis, it was found that as the content of biopolyol in the formulations increased, their thermal stability increased slightly. FTIR analysis confirmed the occurrence of the characteristic absorption bands for rigid PUR-PIR foams (including urethane groups, isocyanate rings, methylene, ester, and ether groups). Furthermore, along with the increase in I_ISO_, an increase in the absorption bands derived from N=C=O, C=O, and isocyanurate rings was observed, which confirmed the formation of PIR structures. To determine the share of PUR and PIR structures in foams, a chemometric analysis based on principal component analysis was performed using the Malinowski spectral isolation algorithm. The PCA confirmed that as the I_ISO_ increased, the proportion of relative PIR concentration increased. For I_ISO_ = 200, it was about 48%, and for I_ISO_ = 300, it was about 65%. 

## Figures and Tables

**Table 1:** Selected properties of polyols applied for the preparation of PUR-PIR foams.

Polyol	L_OH_, mg KOH/g	η, mPa s	ρ, g/cm^3^	H_2_O Content, wt%
LB	650 *	2236	1.21	0.2
Rokopol^®^RF551	440 *	3000–5000	1.06	0.1

*-determined experimentally.

**Table 2:** Formulations of prepared polyurethane-polyisocyanurate (PUR-PIR) foams. DBTDL, dibutyltin dilaurate; TCPP, Trichloropropyl phosphate; pMDI, polymeric methylene diphenyl-4,4’-diisocyanate.

Component	Foam Symbol											
	200_LB0	200_LB10	200_LB20	200_LB30	250_LB0	250_LB10	250_LB20	250_LB30	300_LB0	300_LB10	300_LB20	300_LB30
	Content, pbw											
RF551	100	90	80	70	100	90	80	70	100	90	80	70
LB	0	10	20	30	0	10	20	30	0	10	20	30
AC	0.5	0.5	0.5	0.5	0.5	0.5	0.5	0.5	0.5	0.5	0.5	0.5
Dabco 15K	0.5	0.5	0.5	0.5	0.75	0.75	0.75	0.75	1.00	1.00	1.00	1.00
Dabco 33LV	0.5	0.5	0.5	0.5	0.5	0.5	0.5	0.5	0.5	0.5	0.5	0.5
DBTDL	0.5	0.5	0.5	0.5	0.5	0.5	0.5	0.5	0.5	0.5	0.5	0.5
SPC	6	6	6	6	6	6	6	6	6	6	6	6
TCPP	10	10	10	10	10	10	10	10	10	10	10	10
*n*-pentane	12.5	12.5	12.5	12.5	15.0	15.0	15.0	15.0	20.0	20.0	20.0	20.0
Gram equivalents of OH groups	0.784	0.822	0.859	0.897	0.784	0.822	0.859	0.897	0.784	0.822	0.859	0.897
pMDI	203.6	214.7	225.8	237.0	254.5	268.4	282.3	296.2	305.3	322.0	338.7	355.4
I_ISO_	200				250				300			
Apparent density, kg/m^3^	49.2 ± 1.8	50.1 ± 1.1	50.8 ± 1.9	49.6 ± 1.3	53.2 ± 2.0	50.5 ± 1.8	52.4 ± 1.5	51.7 ± 1.4	49.9 ± 2.2	50.6 ± 1.9	51.9 ± 1.4	52.1 ± 1.2

**Table 3:** Rise times and maximum temperatures recorded during the synthesis of PUR-PIR foams.

I_ISO_	Biopolyol Content, wt%	Rise Time, s	T_MAX_ during Foaming, °C
200	0	40.2 ± 0.4	86.8 ± 2.3
	10	40.3 ± 0.3	87.9 ± 3.5
	20	39.5 ± 0.1	138.2 ± 2.8
	30	38.6 ± 0.4	141.6 ± 3.1
250	0	38.3 ± 0.3	82.4 ± 2.0
	10	37.7 ± 0.2	86.3 ± 2.4
	20	37.5 ± 0.5	133.1 ± 3.4
	30	35.7 ± 0.5	134.6 ± 2.1
300	0	37.4 ± 0.3	71.4 ± 2.0
	10	35.8 ± 0.6	85.2 ± 2.5
	20	34.5 ± 0.4	128.0 ± 3.4
	30	33.3 ± 0.5	130.7 ± 3.2

**Table 4:** Morphological properties of PUR-PIR foams.

Foam Symbol	Average Cell Diameter, µm	Average Cell Volume, mm^3^·10^−3^	Porosity, %
200_LB0	117 ± 21	8.9 ± 2.1	85
200_LB30	108 ± 22	6.5 ± 1.8	81
250_LB0	115 ± 23	8.3 ± 1.6	83
250_LB30	95 ± 16	5.4 ± 2.0	80
300_LB0	131 ± 25	11.0 ± 1.2	84
300_LB30	124 ± 17	7.2 ± 1.6	83

**Table 5:** 3D images of PUR-PIR foams.

Foam Structure	Volume Distribution	Scale, mm^3^ ·10^−3^	
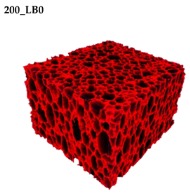	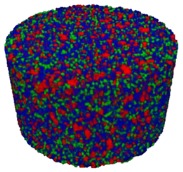		**26–50**
			**13–25**
			**7–12**
			**0–6**
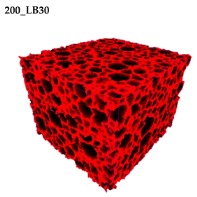	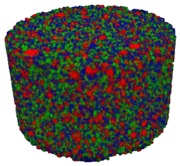		**26–50**
			**13–25**
			**7–12**
			**0–6**
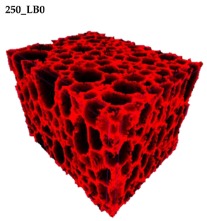	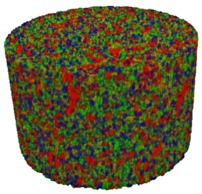		**26–50**
			**13–25**
			**7–12**
			**0–6**
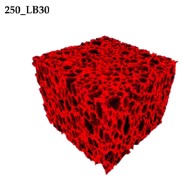	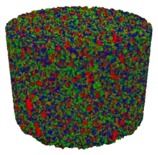		**26–50**
			**13–25**
			**7–12**
			**0–6**
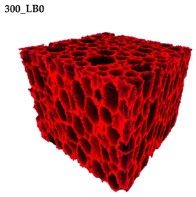	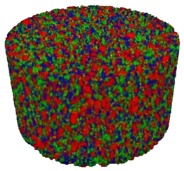		**26–50**
			**13–25**
			**7–12**
			**0–6**
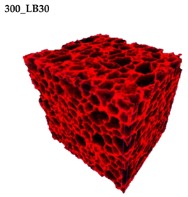	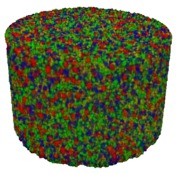		**26–50**
			**13–25**
			**7–12**
			**0–6**

**Table 6:** Thermal insulation properties of the obtained foams.

Foam Symbol	Thermal Conductivity Coefficient, mW/m·K	Thermal Resistance (d = 0.02m), m^2^·K/W
200_LB0	26.98 ± 0.14	0.741
200_LB10	25.33 ± 0.15	0.790
200_LB20	24.74 ± 0.21	0.808
200_LB30	24.69 ± 0.12	0.810
250_LB0	26.15 ± 0.20	0.765
250_LB10	25.38 ± 0.18	0.788
250_LB20	25.25 ± 0.17	0.792
250_LB30	24.91 ± 0.19	0.803
300_LB0	26.09 ± 0.22	0.767
300_LB10	25.44 ± 0.20	0.786
300_LB20	25.11 ± 0.21	0.796
300_LB30	24.83 ± 0.19	0.806

**Table 7:** Physical and mechanical properties of PUR-PIR foams.

Foam Symbol	Sol Fraction Content, wt%	Compressive Strength, kPa			T_g_, °C
		Perpendicular to the Rise Direction	Parallel to the Rise Direction	Anisotropy, %	
200_LB0	2.7 ± 0.7	163 ± 9	394 ± 12	2.42	154
200_LB10	2.2 ± 0.2	176 ± 10	410 ± 8	2.33	200
200_LB20	1.5 ± 0.3	229 ± 8	430 ± 11	1.88	204
200_LB30	1.3 ± 0.7	287 ± 11	446 ± 8	1.55	210
250_LB0	1.2 ± 0.2	168 ± 7	368 ± 6	2.19	213
250_LB10	1.4 ± 1.1	215 ± 12	400 ± 7	1.86	220
250_LB20	0.6 ± 0.1	222 ± 8	414 ± 9	1.86	222
250_LB30	1.5 ± 0.3	243 ± 9	440 ± 8	1.81	225
300_LB0	1.2 ± 1.1	151 ± 7	342 ± 9	2.26	221
300_LB10	0.9 ± 0.2	186 ± 8	366 ± 11	1.97	230
300_LB20	0.8 ± 0.2	190 ± 13	382 ± 14	2.01	232
300_LB30	0.7 ± 0.1	200 ± 7	426 ± 10	2.13	233

**Table 8:** Results of thermogravimetric analysis of PUR-PIR foams.

Foam Symbol	Mass Loss, wt%			T_max_, °C
	2	5	10	
	Temperature, °C			
200_LB0	217.0	250.8	289.2	342.1
200_LB10	224.3	259.7	290.7	347.8
200_LB20	235.8	260.7	290.7	348.8
200_LB30	218.0	251.6	284.8	347.5
250_LB0	198.9	255.9	295.4	344.1
250_LB10	220.4	254.6	289.8	345.2
250_LB20	228.6	264.0	298.5	350.1
250_LB30	239.7	268.8	298.6	349.8
300_LB0	214.6	269.0	300.9	344.2
300_LB10	245.0	275.3	303.7	350.6
300_LB20	242.5	270.5	299	348.1
300_LB30	245.6	273.2	301.5	348.9
